# Supplementary material for: Temperature preference can bias parental genome retention during hybrid evolution
Source: PLoS Genet. 2019 Sep 16;15(9):e1008383. doi: 10.1371/journal.pgen.1008383 (PMC6762194; doi:10.1371/journal.pgen.1008383)
Supplement: S1 Table — (PDF) [file pgen.1008383.s001.pdf]

Table S1: Mutations in cold-evolved *S. cerevisiae* diploid populations

Population	Location	Gene(s)	Mutation
**P7**	chrIV:1323639	*BNA7*	coding-nonsynonymous : I201L
	chrVII:469931	*PDR1*	coding-nonsynonymous : I790F
	chrXVI:166971	*SGE1*	5’ upstream
	chrXVI:933898	*YPR197C*	coding-nonsynonymous: *188Y
	chrXIII:1-221882	Includes *PHO84*	LOH, multiple tract lengths, loss of S288C allele
**P7F**	chrIV:1323639	*BNA7*	coding-nonsynonymous: I201L
	chrXVI:933898	*YPR197C*	coding-nonsynonymous: *188Y
**P8F**	chrIV:1323639	*BNA7*	coding-nonsynonymous: I201L
	chrXVI:167064	*TPK2*	coding-nonsynonymous: P270R
	chrXVI:933898	*YPR197C*	coding-nonsynonymous: *188Y
	chrXIII:1-86512	Includes *PHO84*	LOH: loss of S288C allele
**P9**	chrXIII: 1-221882	Includes *PHO84*	LOH, multiple tract lengths, loss of S288C allele
**P9F**	chrXIII: 1-76194	Includes *PHO84*	LOH: loss of S288C allele
**P10**	chrXIII: 1-221882	Includes *PHO84*	LOH, multiple tract lengths, loss of S288C allele
**G3F**	chrIV: 971450	*RKM4*	coding-nonsynonymous: E9Q
	chrIV: 1214351	*YPR1*	coding-nonsynonymous: T150P
	chrXVI: 166845	*TPK2*	coding-nonsynonymous: E197A
	chrXVI:24965-376139		LOH: loss of S288C
**G4**	chrXVI: 166595	*TPK2*	coding-nonsynonymous: H114N
	chrIX: 1- 439888		CNV: whole chromosome amplification of S288C
	chrV: 374386-576874		LOH: loss of S288C
**G4F**	chrIV: 857504	*RAV2*	coding-nonsynonymous: F150L
	chrV: 374386-576874		LOH: loss of S288C
	chrIX: 1- 439888		CNV: whole chromosome amplification of S288C
	chrXIII: 742394	*RNA1*	coding-nonsynonymous: N114N
	chrXVI: 166595	*TPK2*	coding-nonsynonymous: H114N
**G6**	chrII:211737	*HIR1*	coding-nonsynonymous: G695indel
	chrVI: 71469	*RIM15*	coding-nonsynonymous: P987S
	chrVII: 624765	*YGR067C*	coding-nonsynonymous: Y8N
	chrXII:1-388272		LOH: loss of GRF167 allele
	chrXII:537901-1078177		LOH: loss of S288C allele
	chrXII:202237	*RPL15A*	coding-synonymous: S118S
	chrXV:587229	*YOR139C*	coding-synonymous: H38H
	chrXV:587229	*SFL1*	coding-nonsynonymous: W83*
	chrM:27782	*ATP8*	coding-synonymous: Y39Y
**G6F**	chrVI: 71469	*RIM15*	coding-nonsynonymous: P987S

	chrX: 359553	*GYP6*	coding-nonsynonymous: L42I
	chrXII:537901-1078177		LOH: loss of GRF167 allele
	chrXV: 587229	*YOR139C*	coding-synonymous: H38H
	chrXV: 587229	*SFL1*	coding-nonsynonymous: W83*
**S3**	chrII: 484215	*MRPL36*	coding-nonsynonymous: S97G
	chrV: 277004	*FCY22*	coding-synonymous: L145L
	chrXVI: 166597	*TPK2*	coding-nonsynonymous: H114Q
**S3F**	chrII: 484215	*MRPL36*	coding-nonsynonymous: S97G
	chrIV: 1199893	*ESC2*	coding-synonymous: L237L
	chrVI: 57656	*TUB2*	coding-nonsynonymous: D441N
	chrVII: 1068908	*IMA1*	coding-synonymous: F28F
	chrXIII: 29268	*NDI1*	coding-synonymous: P180P
	chrXIV: 139482	*LYP1*	coding-synonymous: S311S
	chrXVI: 166597	*TPK2*	coding-nonsynonymous: H114Q
	chrXVI: 340228	*CAR1*	coding-synonymous: V95V
**S4F**	chrII: 484215	*MRPL36*	coding-nonsynonymous: S97G
	chrXVI: 166596	*TPK2*	coding-nonsynonymous: H114R
	chrVI: 45080	*OTU1*	coding-nonsynonymous: P161A
	chrXV: 624828	*SLP1*	coding-nonsynonymous: G34S
	chrXV: 810716	*TRE2*	5'-upstream
	chrXVI: 166971	*TPK2*	coding-nonsynonymous: T239R
	chrXVI: 176208	*APL5*	5'-upstream
**S5F**	chrII: 1- 813184		CNV: whole chromosome amplification of GRF167 allele
**S6**	chrII: 528651	*MAK5*	coding-nonsynonymous: E112V
	chrX: 419920-745751		Segmental amplification of GRF167 allele
	chrXII: 537901-1078177		LOH: loss of S288C allele
	chrXV: 810716	*TRE2*	5'-upstream
	chrXVI:1- 948066		CNV: whole chromosome amplification of GRF167 allele
	chrXVI: 166595	*TPK2*	coding-nonsynonymous: H114Y
**S6F**	chrX: 424951-745751		CNV: segmental amplification of GRF167 allele
	chrXV: 810716	*TRE2*	5'-upstream

LOH: loss of heterozygosity; CNV: copy number variant. Breakpoints of CNV and LOH are approximate. Note, because of population sequencing, it is not possible to definitively determine whether CNV are also accompanied by LOH.
