# Supplementary material for: Temperature preference can bias parental genome retention during hybrid evolution
Source: PLoS Genet. 2019 Sep 16;15(9):e1008383. doi: 10.1371/journal.pgen.1008383 (PMC6762194; doi:10.1371/journal.pgen.1008383)
Supplement: S2 Table — (PDF) [file pgen.1008383.s002.pdf]

Table S2: Mutations in evolved *S. uvarum* diploid populations

Population	Location	Gene(s)	Mutation
**G1**	chrII:1-1289935		CNV: whole chromosome amplification
	chrX: 1-1016005		CNV: whole chromosome amplification
	chrXII: 1-445542		CNV: whole chromosome amplification
**S2**	*S. uvarum* chrXII: 111565	*CYR1*	coding-nonsynonymous: R1343C

CNV: copy number variant. No mutations were detected in P11, P12, G2, S1.
