# Supplementary material for: Temperature preference can bias parental genome retention during hybrid evolution
Source: PLoS Genet. 2019 Sep 16;15(9):e1008383. doi: 10.1371/journal.pgen.1008383 (PMC6762194; doi:10.1371/journal.pgen.1008383)
Supplement: S3 Table — (PDF) [file pgen.1008383.s003.pdf]

		Number of SNPs and InDels					
		Hybrid			Parental species		
		S. cer	*S. uva*	Total	*S. cer*	*S. uva*	Total
15°C	Populations	12	7	19	19	1	20
	Floc. Clones	1	1	2	34	0	34
30°C	Clones	16	14	40	7	12	19
