# Supplementary material for: Temperature preference can bias parental genome retention during hybrid evolution
Source: PLoS Genet. 2019 Sep 16;15(9):e1008383. doi: 10.1371/journal.pgen.1008383 (PMC6762194; doi:10.1371/journal.pgen.1008383)
Supplement: S4 Table — (PDF) [file pgen.1008383.s004.pdf]

Table S4: Strain list

Strain	Identifier	Genotype	Species	Notes	Strain background
YMD139		*lys2Δ0*/*LYS2 ura3-167/URA3 MATa*/*MATα*	cer	Ancestor of *S. cerevisiae* evolved diploids (high Ty)	GRF167 x S288C
YMD140		*lys2Δ0*/*LYS2 ura3-167/URA3 MATa*/*MATα*	cer	Ancestor of *S. cerevisiae* evolved diploids	GRF167 x S288C
YMD129		*HOΔ::*kanMX/*HOΔ::*kanMX *lys2-1/ LYS2 ura3-167/URA3 MATa*/*MATα*	hybrid	Ancestor of evolved hybrids	GRF167 x CBS7001
YMD130		*HOΔ::*kanMX/*HOΔ::*kanMX *lys2-1/ LYS2 ura3-167/URA3 MATa/MATα* (high Ty)	hybrid	Ancestor of evolved hybrids (high Ty)	GRF167 x CBS7001
YMD366		*HOΔ::*kanMX /*HOΔ::*kanMX *lys2-1/ LYS2 ura3Δ::*cloNAT/*URA3 MATa/MATα*	uva	Ancestor of *S. uvarum* evolved diploids	CBS7001
	G1		uva	Glucose limitation, 15°C	YMD366
	G2		uva	Glucose limitation, 15°C	YMD366
	G3F		cer	Glucose limitation, 15°C	YMD139
	G4		cer	Glucose limitation, 15°C	YMD139
	G4F		cer	Glucose limitation, 15°C	YMD139
	G6		cer	Glucose limitation, 15°C	YMD140
	G6F		cer	Glucose limitation, 15°C	YMD140
	G7		hybrid	Glucose limitation, 15°C	YMD130
YMD3928	G7 c1		hybrid	Glucose limitation, 15°C	YMD130
YMD3929	G7 c2		hybrid	Glucose limitation, 15°C	YMD130
	G8		hybrid	Glucose limitation, 15°C	YMD130
YMD3930	G8 c1		hybrid	Glucose limitation, 15°C	YMD130
YMD3931	G8 c2		hybrid	Glucose limitation, 15°C	YMD130
	G9		hybrid	Glucose limitation, 15°C	YMD129
YMD3932	G9 c1		hybrid	Glucose limitation, 15°C	YMD129
YMD3933	G9 c2		hybrid	Glucose limitation, 15°C	YMD129
	G10		hybrid	Glucose limitation, 15°C	YMD129

YMD3934	G10 c1		hybrid	Glucose limitation, 15°C	YMD129
	S1		uva	Sulfate limitation, 15°C	YMD366
	S2		uva	Sulfate limitation, 15°C	YMD366
	S3		cer	Sulfate limitation, 15°C	YMD139
	S3F		cer	Sulfate limitation, 15°C	YMD139
	S4F		cer	Sulfate limitation, 15°C	YMD139
	S5F		cer	Sulfate limitation, 15°C	YMD140
	S6		cer	Sulfate limitation, 15°C	YMD140
	S6F		cer	Sulfate limitation, 15°C	YMD140
	S7		hybrid	Sulfate limitation, 15°C	YMD130
YMD3935	S7 c1		hybrid	Sulfate limitation, 15°C	YMD130
YMD3936	S7 c2		hybrid	Sulfate limitation, 15°C	YMD130
	S8		hybrid	Sulfate limitation, 15°C	YMD130
YMD3937	S8 c1		hybrid	Sulfate limitation, 15°C	YMD130
YMD3938	S8 c1		hybrid	Sulfate limitation, 15°C	YMD130
	S9		hybrid	Sulfate limitation, 15°C	YMD129
YMD3939	S9 c1		hybrid	Sulfate limitation, 15°C	YMD129
	S10		hybrid	Sulfate limitation, 15°C	YMD129
YMD3940	S10 c1		hybrid	Sulfate limitation, 15°C	YMD129
	P1		hybrid	Phosphate limitation, 15°C	YMD130
YMD3950	P1 c1		hybrid	Phosphate limitation, 15°C	YMD130
YMD3951	P1 c2		hybrid	Phosphate limitation, 15°C	YMD130
	P2		hybrid	Phosphate limitation, 15°C	YMD130
YMD3941	P2 c1		hybrid	Phosphate limitation, 15°C	YMD130
YMD3942	P2 c2		hybrid	Phosphate limitation, 15°C	YMD130
	P3		hybrid	Phosphate limitation, 15°C	YMD130
YMD3948	P3 c3		hybrid	Phosphate limitation, 15°C	YMD130
YMD3949	P3 c5		hybrid	Phosphate limitation,	YMD130

				15°C	
	P4		hybrid	Phosphate limitation, 15°C	YMD130
YMD3943	P4 c1		hybrid	Phosphate limitation, 15°C	YMD130
YMD3944	P4 c2		hybrid	Phosphate limitation, 15°C	YMD130
	P5		hybrid	Phosphate limitation, 15°C	YMD129
YMD3945	P5 c1		hybrid	Phosphate limitation, 15°C	YMD129
YMD3946	P5 c2		hybrid	Phosphate limitation, 15°C	YMD129
	P6		hybrid	Phosphate limitation, 15°C	YMD129
YMD3947	P6 c1		hybrid	Phosphate limitation, 15°C	YMD129
	P7		cer	Phosphate limitation, 15°C	YMD140
	P7F		cer	Phosphate limitation, 15°C	YMD140
	P8F		cer	Phosphate limitation, 15°C	YMD140
	P9		cer	Phosphate limitation, 15°C	YMD139
	P9F		cer	Phosphate limitation, 15°C	YMD139
	P10		cer	Phosphate limitation, 15°C	YMD139
	P11		uva	Phosphate limitation, 15°C	YMD366
	P12		uva	Phosphate limitation, 15°C	YMD366
YMD30	P1-30°		hybrid	Phosphate limitation, 30°C	YMD130
YMD39	P2-30°		hybrid	Phosphate limitation, 30°C	YMD130
YMD90	P3-30°		hybrid	Phosphate limitation, 30°C	YMD129
YMD101	P5-30°		hybrid	Phosphate limitation, 30°C	YMD130
YMD741	P4-30°		hybrid	Phosphate limitation, 30°C	YMD129
YMD744	P6-30°		hybrid	Phosphate limitation, 30°C	YMD129
YMD3353		*HOΔ::*HYGb/*HOΔ::*kanMX *lys2-1*/ *LYS2* *ura3Δ::*cloNAT/*ura3-167* *PHO84*Δ::kanMX/*PHO84* *MATa*/*MATα* *+pIL37-PHO84-URA3*	hybrid	2+ preferred *S. cerevisiae* alleles at *PHO84*	
YMD3354		*HOΔ::*HYGb/*HOΔ::*kanMX *lys2-1*/ *LYS2* *ura3Δ::*cloNAT/*ura3-167* *PHO84*Δ::kanMX/*PHO84*	hybrid	1 preferred *S. cerevisiae* allele (empty plasmid control for	

		MATa/*MATα* +*pIL37-URA3*		YMD3353)	
YMD3892		*ura3*-*167*/*URA3 lys2*-1/*LYS2 ScerPHO84Δ::SuvaPHO84/ PHO84*	hybrid	2 *S. uvarum* alleles at *PHO84*	
YMD4246		*ura3-167/URA3 ScerPHO84Δ::SuvaPHO84/ ScerPHO84Δ::SuvaPHO84*	cer	2 *S. uvarum* alleles at *PHO84*	
YMD1459		*HO*Δ*::*kanMX-GFP/*HO MATa/MATα*	cer	GFP competitor strain for *S. cerevisiae* diploid	
YMD3357		*HO*Δ*::*natMX-GFP/*HO lys2*-*1*/ *LYS2 ura3*-*167*/*URA3 MATa/MATα*	hybrid	GFP competitor strain for hybrids with strain background YMD129	
YMD3358		*HO*Δ*::*natMX-GFP/*HO lys2*-*1*/ *LYS2 ura3*-*167*/*URA3 MATa/MATα* (high Ty)	hybrid	GFP competitor strain for hybrids with strain background YMD130	

Populations are identified by nutrient (G: glucose-limitation, P: phosphate-limitation, and S: sulfate-limitation) and the number indicates its derivation from independent populations. "F" denotes a flocculent population isolated from the culture. The strain number in the Dunham lab strain database is listed for each strain (clone) used in fitness competition assays. No strain number is listed for evolved populations, which were archived in glycerol twice a week throughout the experiment.
