# Supplementary material for: Temperature preference can bias parental genome retention during hybrid evolution
Source: PLoS Genet. 2019 Sep 16;15(9):e1008383. doi: 10.1371/journal.pgen.1008383 (PMC6762194; doi:10.1371/journal.pgen.1008383)
Supplement: S5 Table — (PDF) [file pgen.1008383.s005.pdf]

Table S5: Competitive fitness of hybrids at two temperatures

Population Identifier	Original evolution conditions	Competitive Fitness at 15°C (+/- 95% C.I.)	Competitive Fitness at 30°C (+/- 95% C.I.)
**G7-15°**	Glucose limitation, 15°C	C1: -0.66<br>C2: 6.37 (+/-50.27)	C1: 1.86<br>C2: 6.63 (+/-17.66)
**G8-15°**	Glucose limitation, 15°C	C1: 2.65 (+/-10.79)<br>C2: 14.83 (+/-46.16)	C2: 2.07 (+/-49.67)
**G9-15°**	Glucose limitation, 15°C	C1: 6.40 (+/-3.55)<br>C2: 11.76	C1: 10.10 (+/-35.96)<br>C2: -2.45 (+/-21.37)
**G10-15°**	Glucose limitation, 15°C	C1: 5.14 (+/-0.36)	C1: 13.06 (+/-58.47)
**S7-15°**	Sulfate limitation, 15°C	C1: 7.43 (+/-3.47)<br>C2: 28.52 (+/-18.04)	C1: 23.66<br>C2: 30.12 (+/-129.57)
**S8-15°**	Sulfate limitation, 15°C	C1: 18.55 (+/-46.41)<br>C2: 30.85 (+/-15.04)	C1: 39.38 (+/-15.94)<br>C2: 37.47 (+/-9.72)
**S9-15°**	Sulfate limitation, 15°C	C1: 7.40 (+/-55.15)	C1: 12.54 (+/-47.03)
**S10-15°**	Sulfate limitation, 15°C	C1: 12.26 (+/-16.05)	C1: 41.01 (+/-51.94)
**P1-15°**	Phosphate limitation, 15°C	C1: 7.38 (+/-1.52)<br>C2: 10.01 (+/-1.96)	C1: -4.81 (+/-5.14)<br>C2: 0.40 (+/-10.07)
**P2-15°**	Phosphate limitation, 15°C	C1: -1.83 (+/-5.08)<br>C2: -1.93(+/-1.16)	C1: -3.82 (+/-8.73)<br>C2: 9.34 (+/-9.70)
**P3-15°**	Phosphate limitation, 15°C	C1: 8.34 (+/-3.67)<br>C2: 5.13 (+/-2.76)	C1: -7.59 (+/-6.58)<br>C2: 0.76 (+/-29.25)
**P4-15°**	Phosphate limitation, 15°C	C1: 0.07 (+/-3.18)<br>C2: -3.50 (+/-44.24)	C1: 14.87 (+/-28.11)<br>C2: -0.58 (+/-32.39)
**P5-15°**	Phosphate limitation, 15°C	C1: 16.46 (+/-11.85)<br>C2: 10.23 (+/-15.04)	C1: 10.48 (+/-14.72)
**P6-15°**	Phosphate limitation, 15°C	C1: 10.09 (+/-7.72)<br>C2: 6.90 (+/-10.01)	C1: 10.49 (+/-18.45)
**P1-30°**	Phosphate limitation, 30°C	21.01	29.18 (+/-17.42)

**P2-30°**	Phosphate limitation, 30°C	14.55 (+/-37.40)	25.34 (+/-3.11)
**P3-30°**	Phosphate limitation, 30°C	5.25 (+/-22.44)	30.03 (+/-54.76)
**P5-30°**	Phosphate limitation, 30°C	-8.34 (+/-3.71)	21.88 (+/-105.77)
**P4-30°**	Phosphate limitation, 30°C	-10.67 (+/-7.47)	18.67 (+/-46.00)
**P6-30°**	Phosphate limitation, 30°C	-1.76 (+/-8.38)	17.17 (+/-29.79)

One or two clones (denoted as C1, C2) were selected from each cold-evolved population and competed against a GFP-tagged ancestor in the nutrient limitation they in which they were evolved at both 15°C and 30°C. Six clones evolved in phosphate limitation at 30°C from Smukowski Heil et al. 2017 were also tested at 15°C and 30°C.
