# Supplementary material for: Temperature preference can bias parental genome retention during hybrid evolution
Source: PLoS Genet. 2019 Sep 16;15(9):e1008383. doi: 10.1371/journal.pgen.1008383 (PMC6762194; doi:10.1371/journal.pgen.1008383)
Supplement: S6 Table — (PDF) [file pgen.1008383.s006.pdf]

Table S6: Filters used in variant calling.

Samtools Filter Parameters

Type	Shorthand	Value
**Mapping quality**	MQ	>30
**Quality score**	QUAL	>50
**Read depth**	DP	>40
**Alternate read count**	DP4[2]+DP4[3]	>4
**Forward read balance**	(DP4[0]+DP4[2])/DP	>0.01
**Reverse read balance**	(DP4[1]+DP4[3])/DP	>0.01

Freebayes Filter Parameters

Type	Shorthand	Value
**Mapping quality of observed alternate alleles**	MQM	>30
**Mapping quality of observed reference alleles**	MQMR	>30
**Quality score**	QUAL	>20
**Read depth**	DP	>40
**Forward strand alternate read count**	SAF	>2
**Reverse strand alternate read count**	SAR	>2
**Forward read balance**	SRF + SAF / DP	>0.01
**Reverse read balance**	SRR + SAR / DP	>0.01

LoFreq Filter Parameters

Type	Shorthand	Value
**Quality score**	QUAL	>20
**Read depth**	DP	>20
**Forward strand alternate read count**	DP4[2]	>2
**Reverse strand alternate read count**	DP4[3]	>2
**Forward read balance**	(DP4[0]+DP4[2])/DP	>0.01
**Reverse read balance**	(DP4[1]+DP4[3])/DP	>0.01
