# Supplementary figures and images for: Temperature preference can bias parental genome retention during hybrid evolution
Source: PLoS Genet. 2019 Sep 16;15(9):e1008383. doi: 10.1371/journal.pgen.1008383 (PMC6762194; doi:10.1371/journal.pgen.1008383)

Figure S1

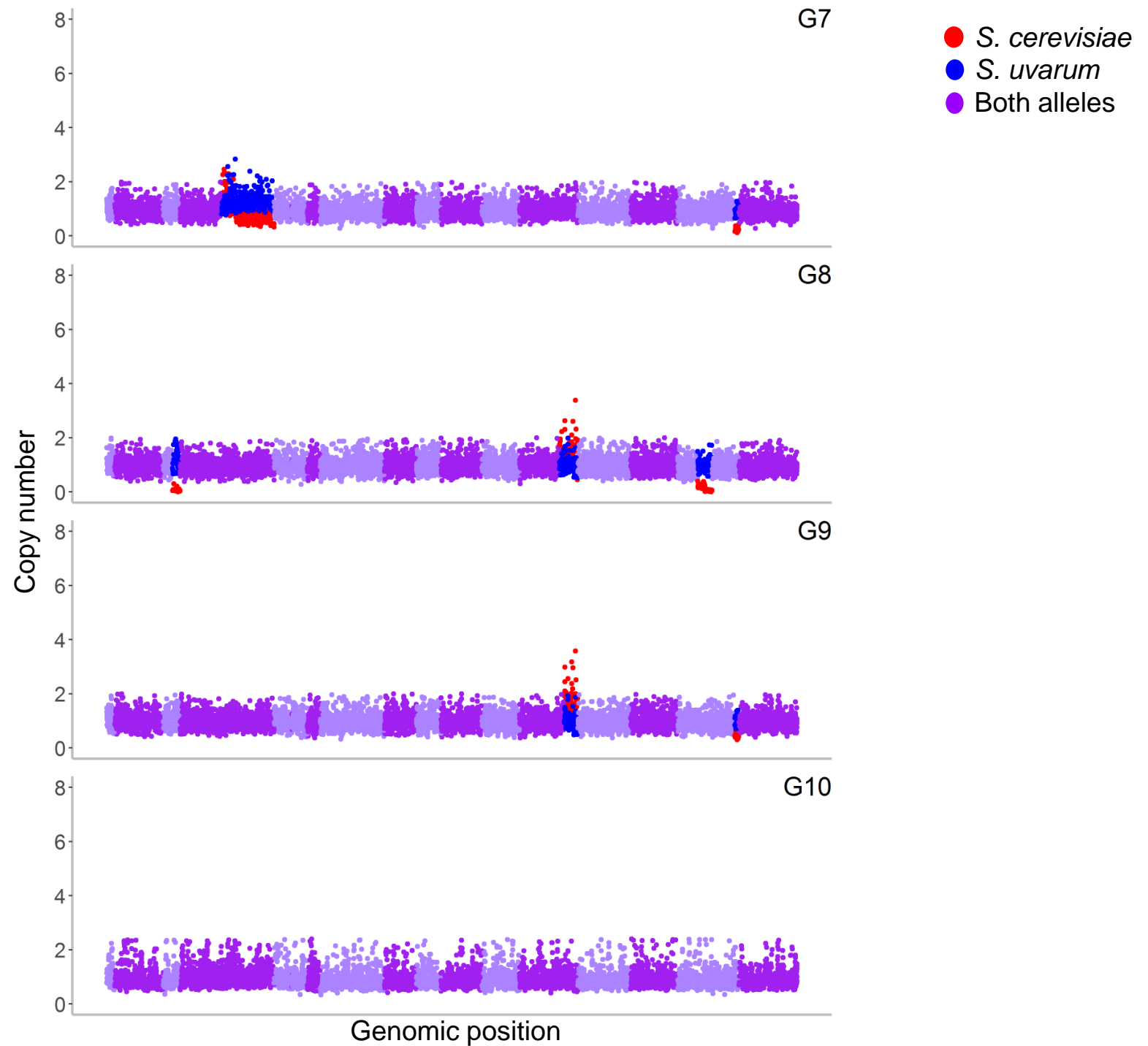

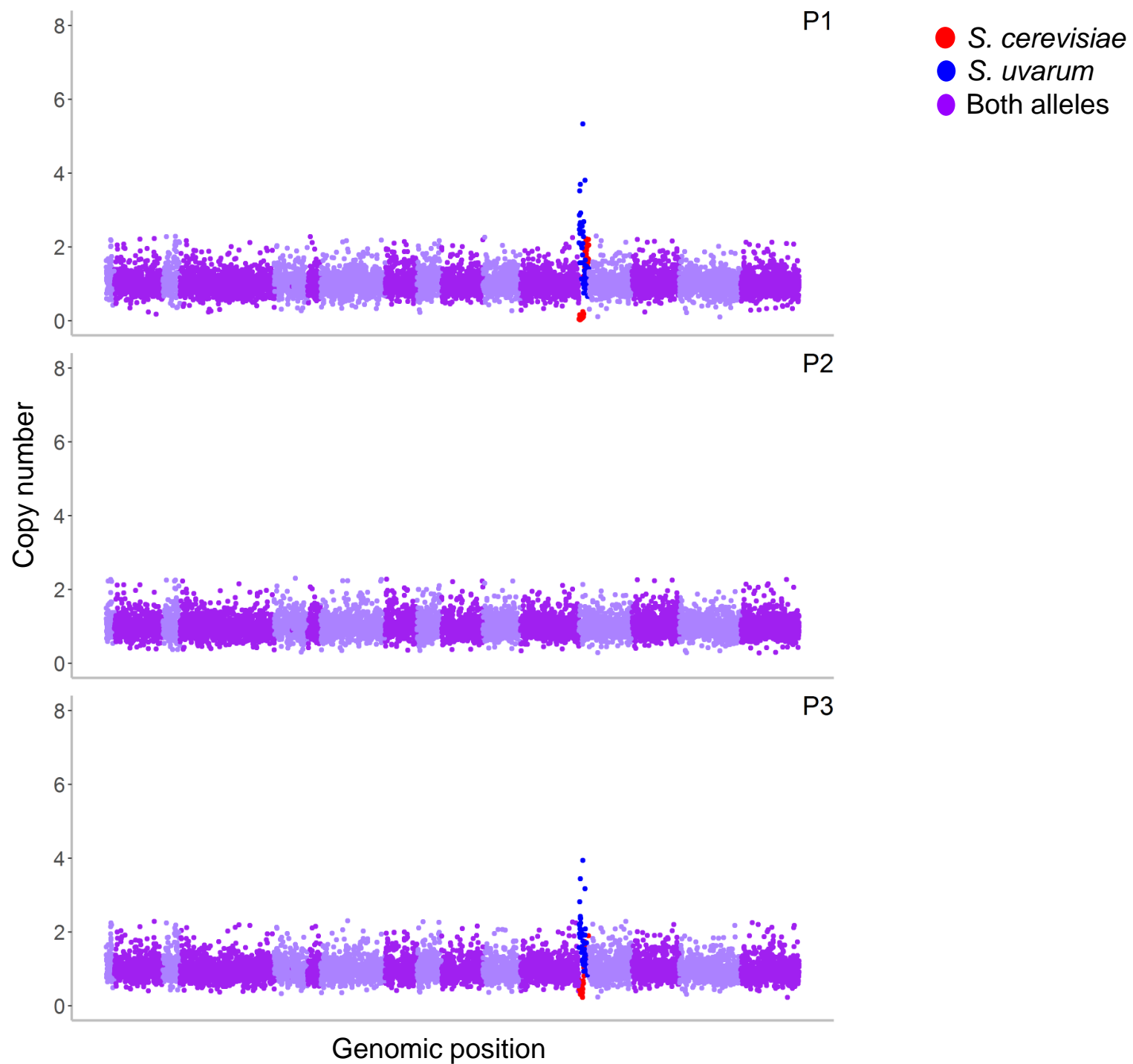

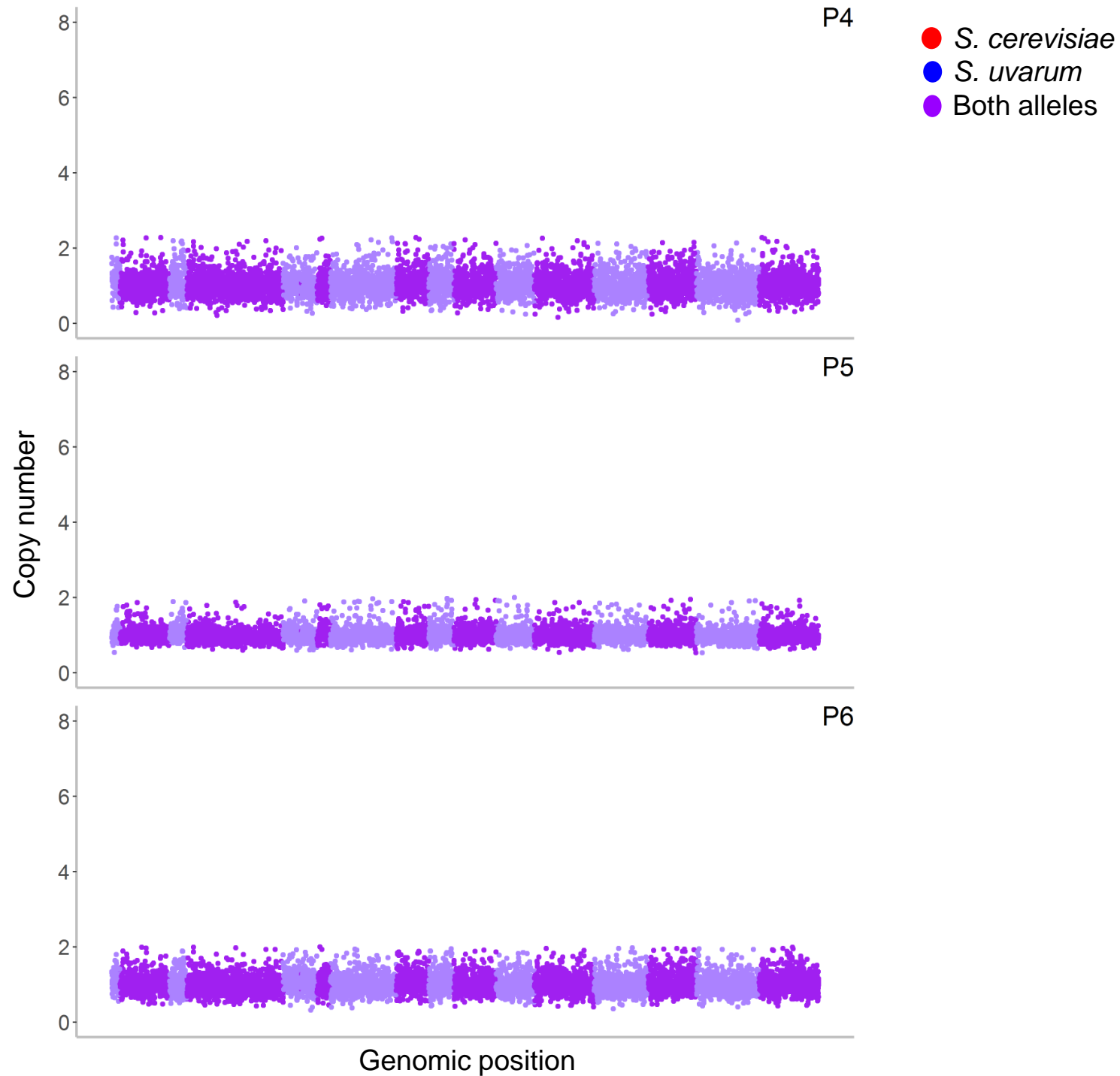

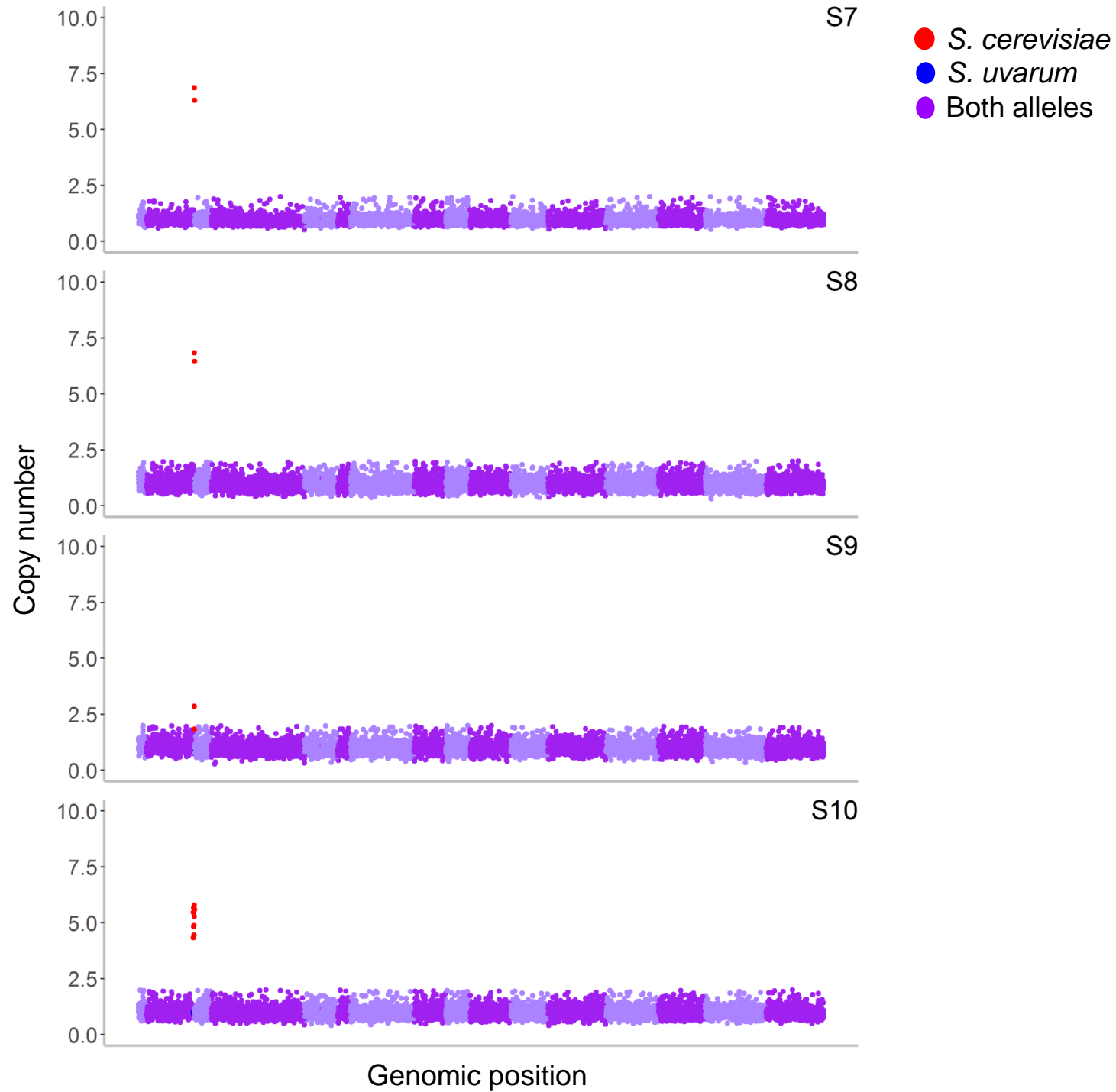

Supplement: S1 Fig — Genome wide copy number is plotted for evolved hybrid populations at 15°C. Nutrient limitation is indicated in the upper right corner (G = glucose, S = sulfate, P = phosphate), numbers indicate independent populations. Purple denotes a region where both alleles are present at a single copy (alternating purple indicates different chromosomes from chrI—chrXVI), blue denotes a S. uvarum change in copy number, red denotes a S. cerevisiae change in copy number. Note, copy number was derived from population sequencing read depth at homologous ORFs. (PDF) [file pgen.1008383.s008.pdf]

Figure S2

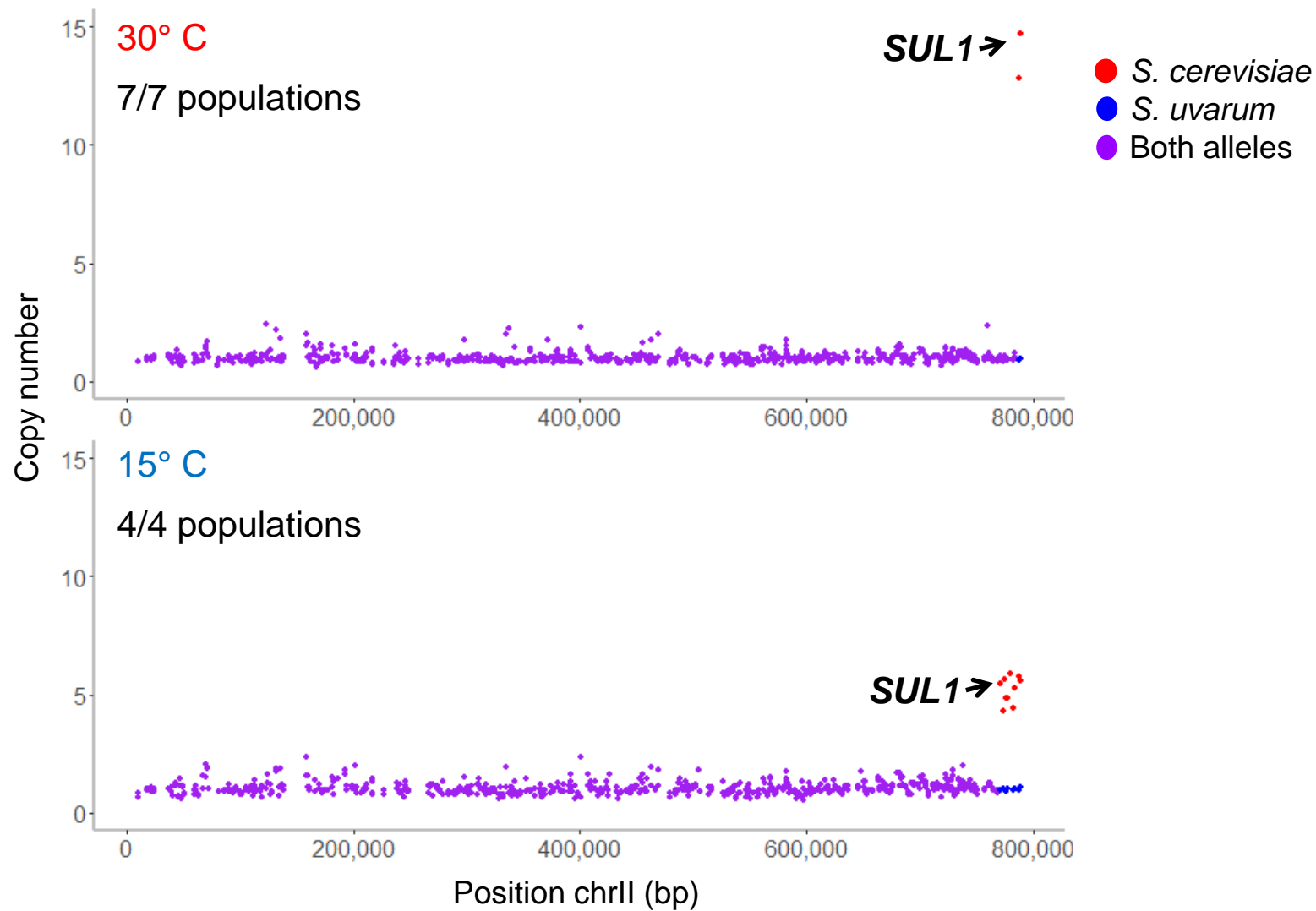

Supplement: S2 Fig — Copy number is plotted across chrII in a representative hybrid clone evolved in sulfate limitation at 30°C and a population evolved in sulfate limitation at 15°C. Copy number was derived from sequencing read depth at homologous ORFs. A region containing the S. cerevisiae allele of the high affinity sulfate transporter SUL1 is amplified in 7/7 populations evolved at 30°C and 4/4 populations evolved at 15°C, suggesting that the locus is not temperature sensitive, and instead that the S. cerevisiae allele is more fit at both temperatures. (PDF) [file pgen.1008383.s009.pdf]

Figure S3

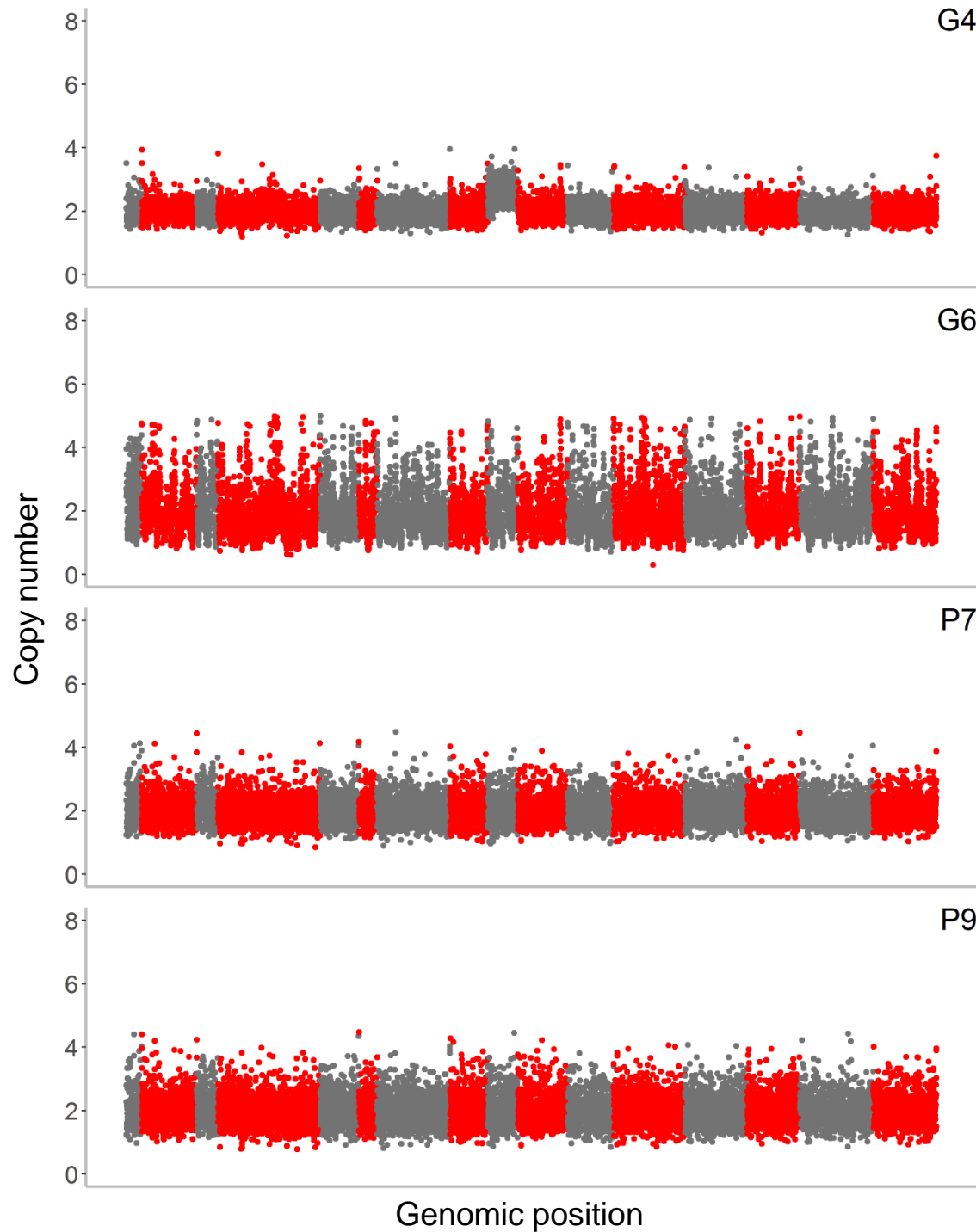

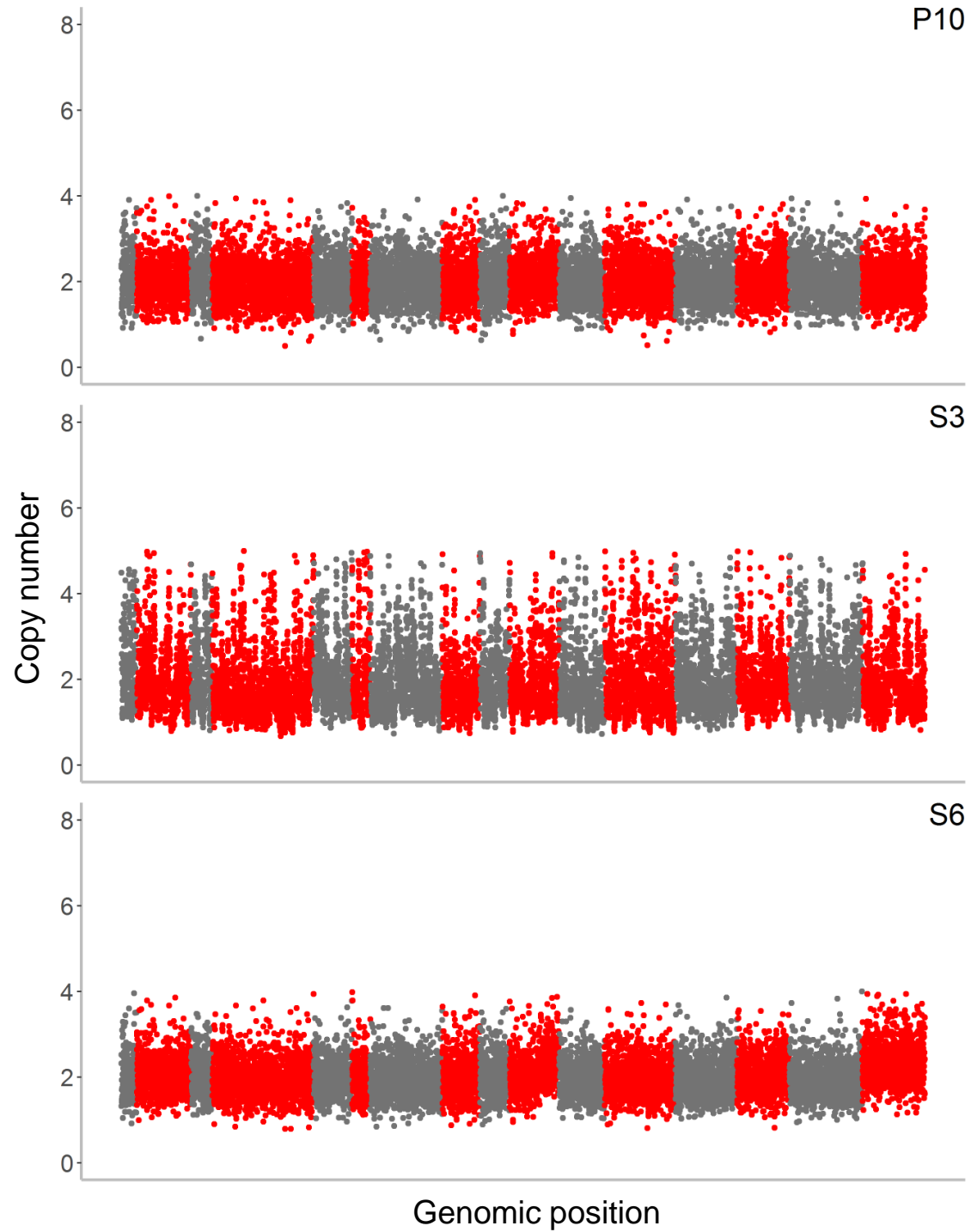

Supplement: S3 Fig — Copy number is plotted across the genome for S. cerevisiae evolved populations. Alternating grey and red indicate different chromosomes (from chrI–chrXVI). Copy number was derived from average population sequencing read depth in 1000 bp intervals. Nutrient limitation is indicated in the upper right corner (G = glucose, S = sulfate, P = phosphate), numbers indicate independent populations. (PDF) [file pgen.1008383.s010.pdf]

Figure S4

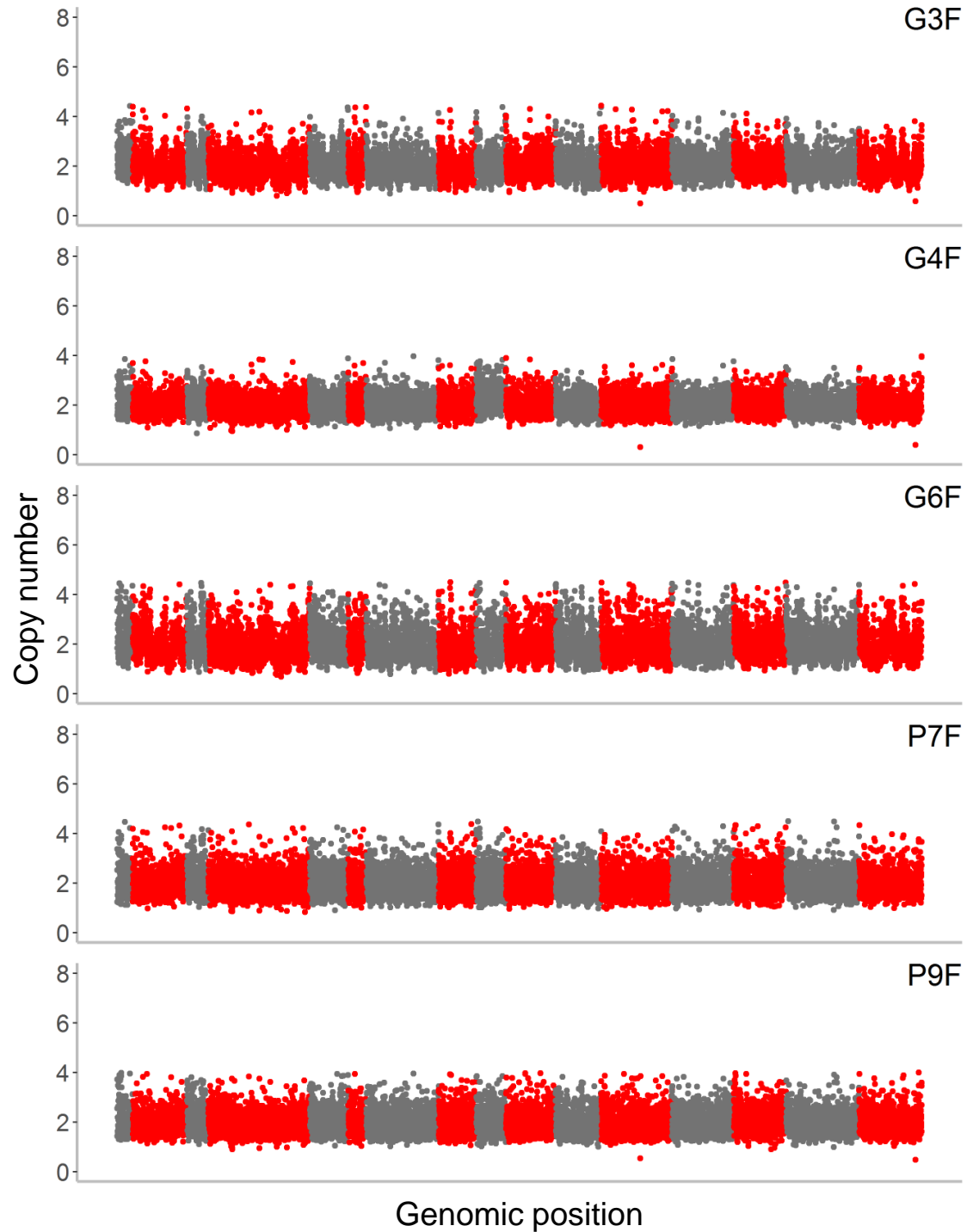

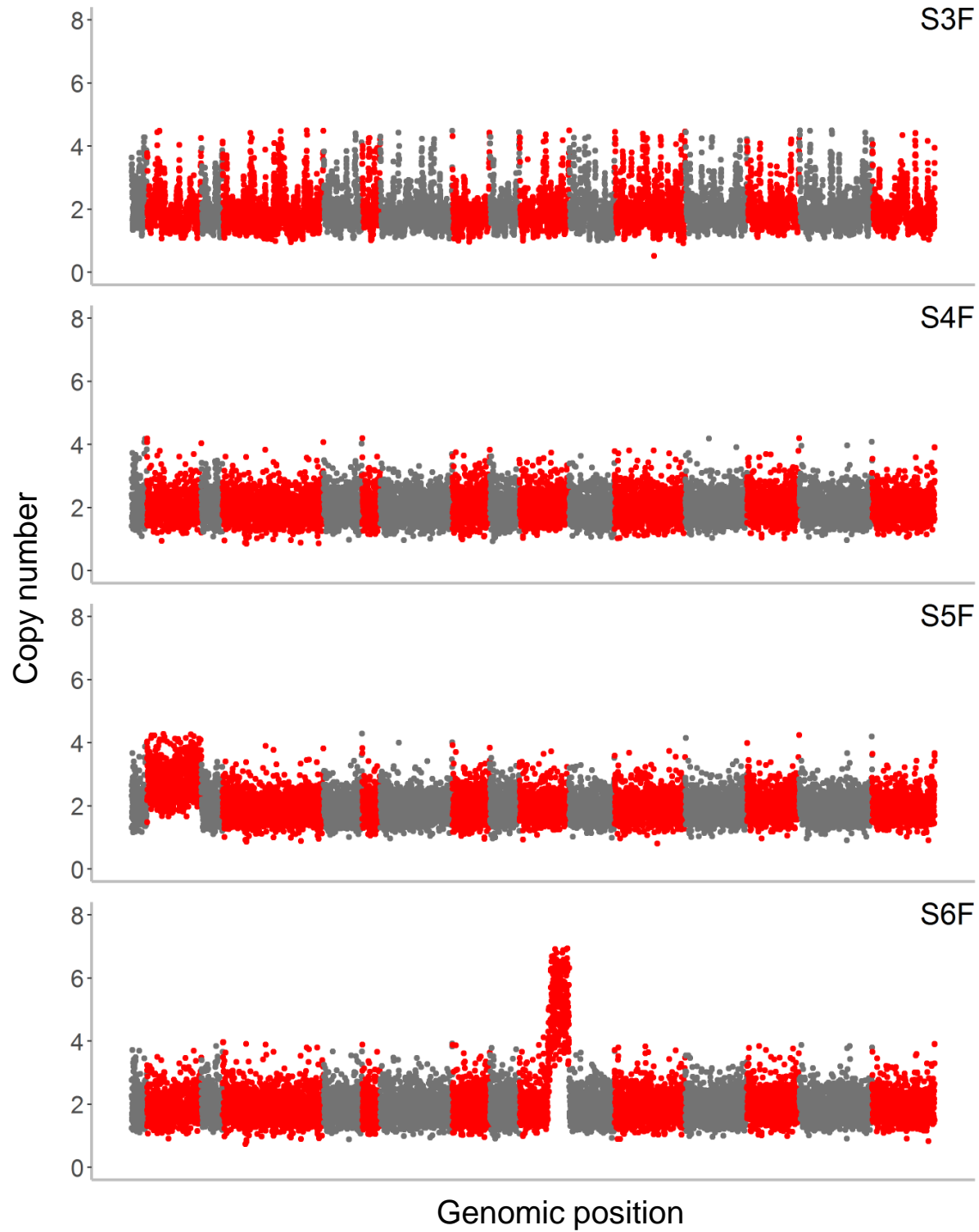

Supplement: S4 Fig — Copy number is plotted across the genome for S. cerevisiae evolved, flocculent populations that were isolated separately from populations dispersed in the culture. Alternating grey and red indicate different chromosomes (from chrI–chrXVI). Copy number was derived from average population sequencing read depth in 1000 bp intervals. Nutrient limitation is indicated in the upper right corner (G = glucose, S = sulfate, P = phosphate), numbers indicate independent populations. (PDF) [file pgen.1008383.s011.pdf]

Figure S5

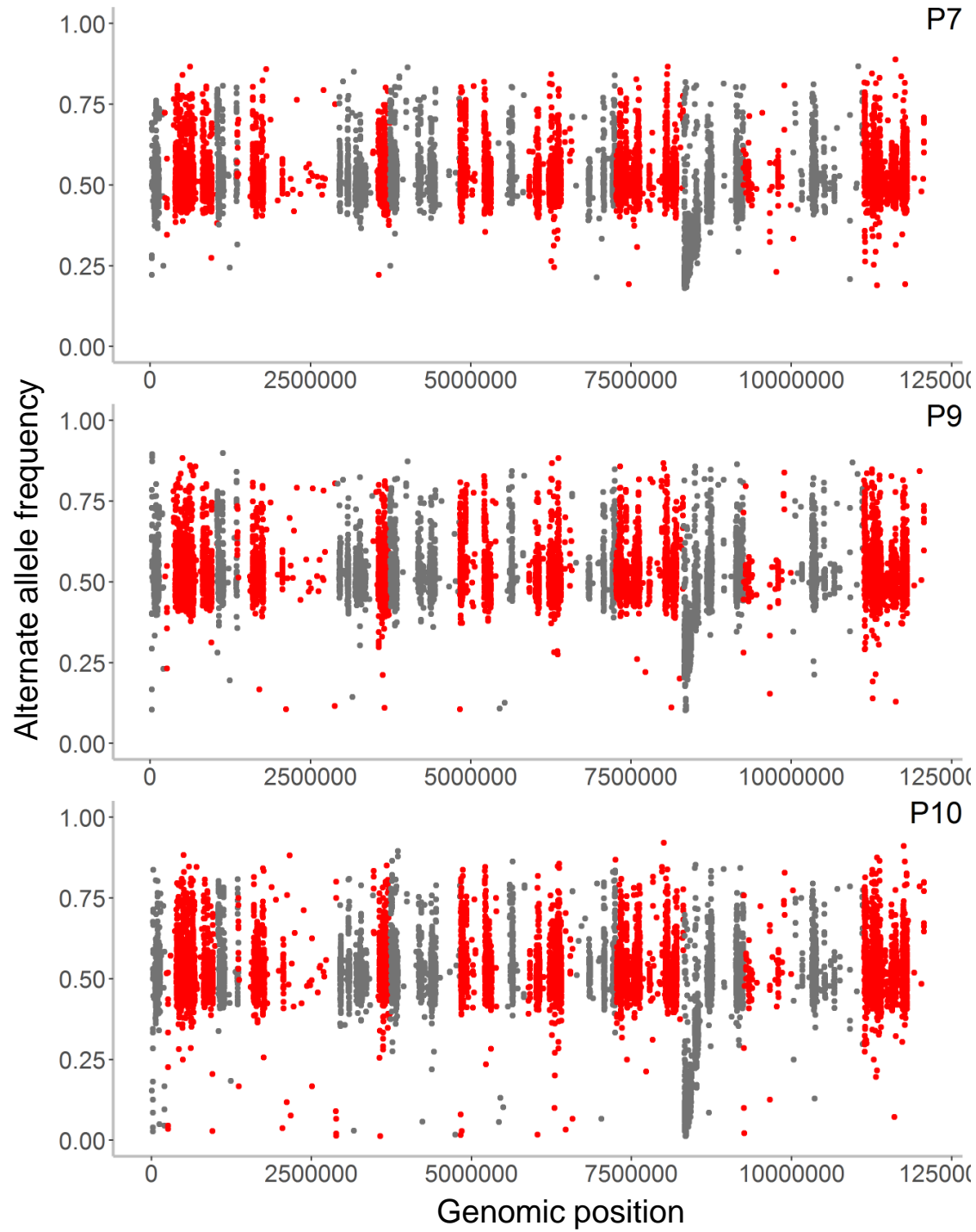

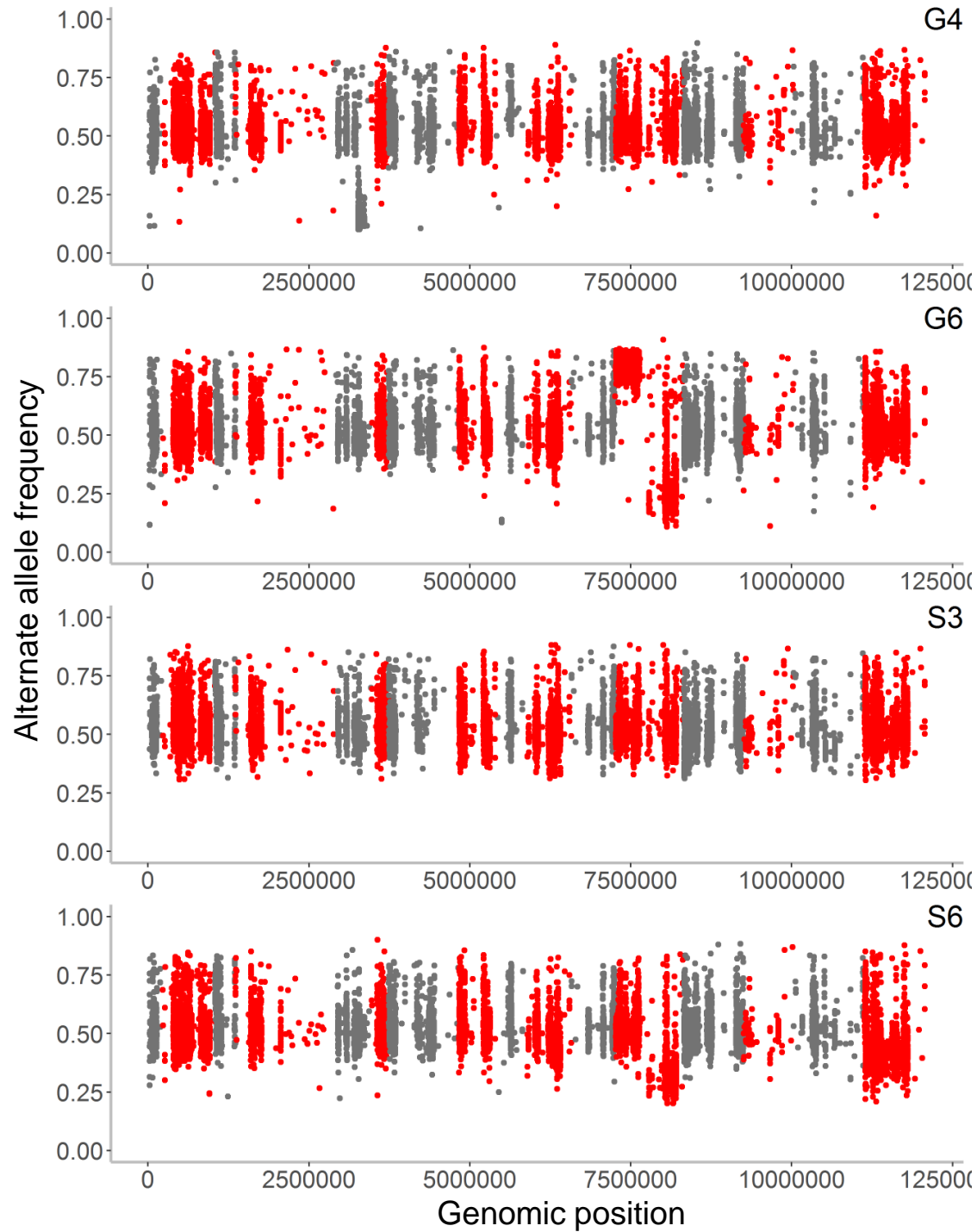

Supplement: S5 Fig — Alternate allele (e.g., non-reference allele) frequency is plotted across the genome for S. cerevisiae evolved populations. The unique pattern of heterozygosity is produced by a strain history of crossing FL100 to S288C to produce GRF167, which was crossed to S288C to produce the diploid strain used here. This produced regions of heterozygosity and regions of homozygosity (regions that appear blank because no alternate allele is called). This also allows the detection of loss of heterozygosity (LOH), where regions that were heterozygous become homozygous for the reference or non-reference allele. Alternating grey and red indicate different chromosomes (from chrI–chrXVI). Nutrient limitation is indicated in the upper right corner (G = glucose, S = sulfate, P = phosphate), numbers indicate independent populations. Note that LOH events are not at fixation in the population, so these events are instead indicated by allele frequencies approaching zero or one. (PDF) [file pgen.1008383.s012.pdf]

Figure S6

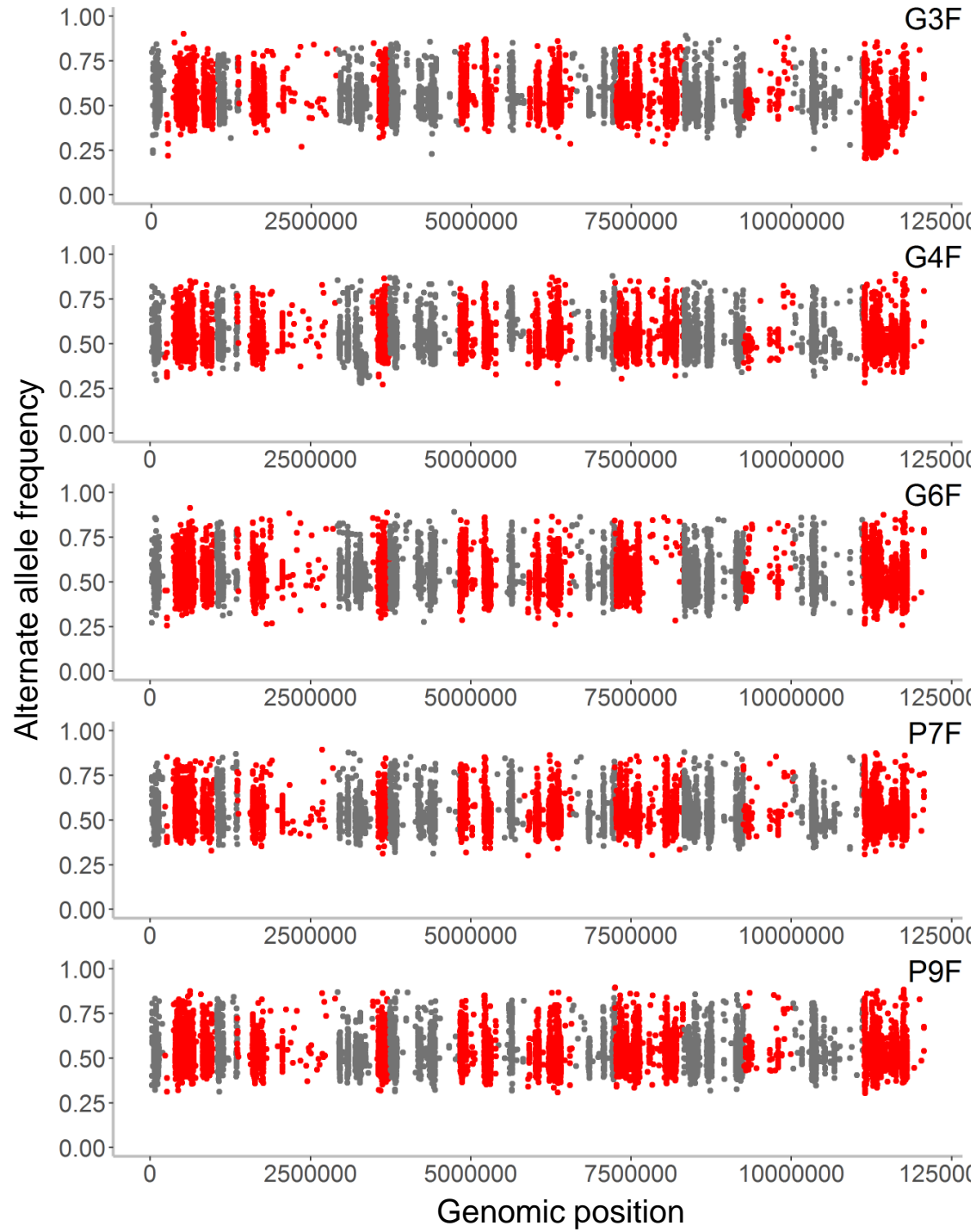

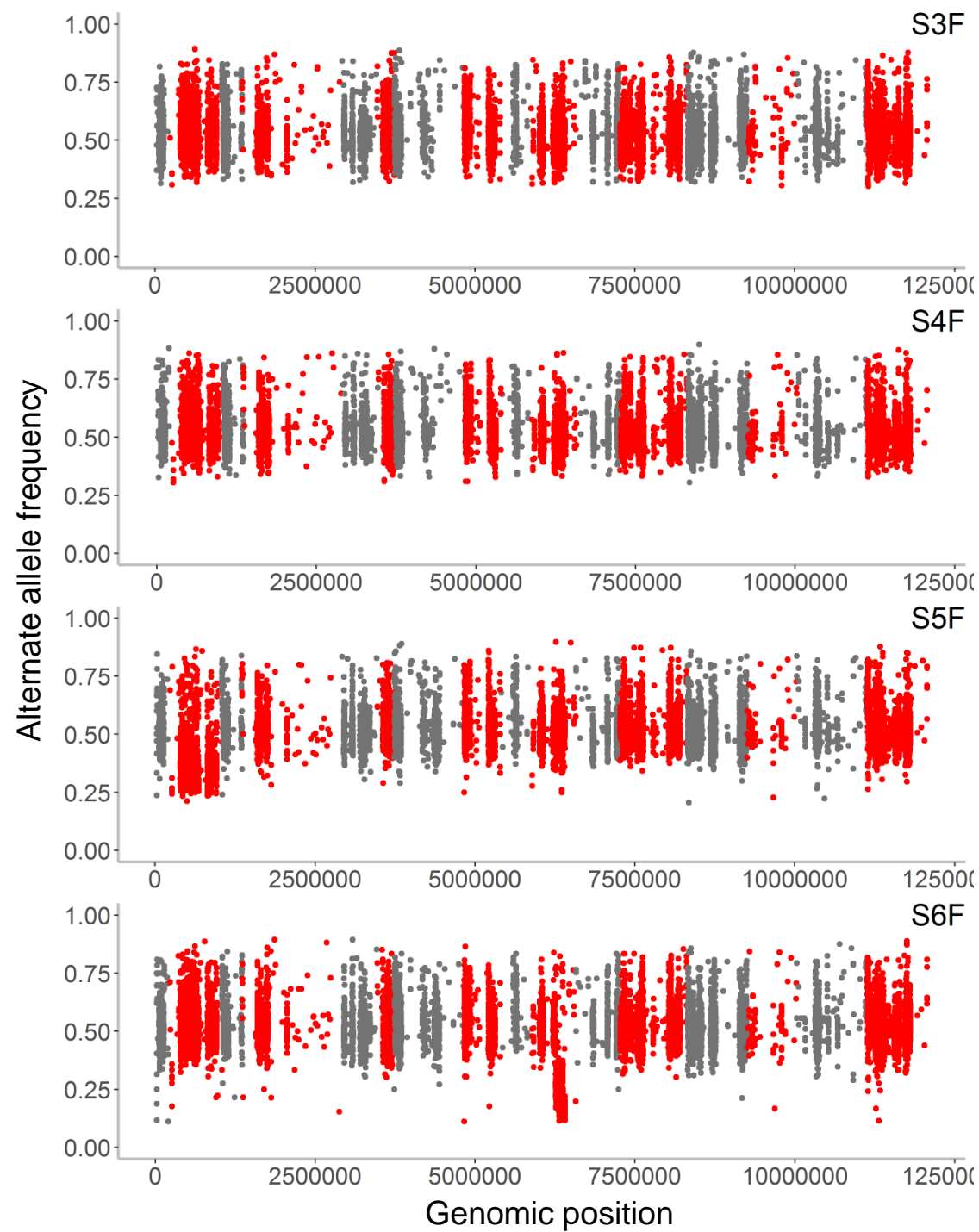

Supplement: S6 Fig — Alternate allele (e.g., non-reference allele) frequency is plotted across the genome for S. cerevisiae evolved, flocculent populations that were isolated separately from populations dispersed in the culture. The unique pattern of heterozygosity is produced by a strain history of crossing FL100 to S288C to produce GRF167, which was crossed to S288C to produce the diploid strain used here. This produced regions of heterozygosity and regions of homozygosity (regions that appear blank because no alternate allele is called). This also allows the detection of loss of heterozygosity (LOH), where regions that were heterozygous become homozygous for the reference or non-reference allele. Alternating grey and red indicate different chromosomes (from chrI–chrXVI). Nutrient limitation is indicated in the upper right corner (G = glucose, S = sulfate, P = phosphate), numbers indicate independent populations. Note that LOH events are not at fixation in the population, so these events are instead indicated by allele frequencies approaching zero or one. (PDF) [file pgen.1008383.s013.pdf]

Figure S7

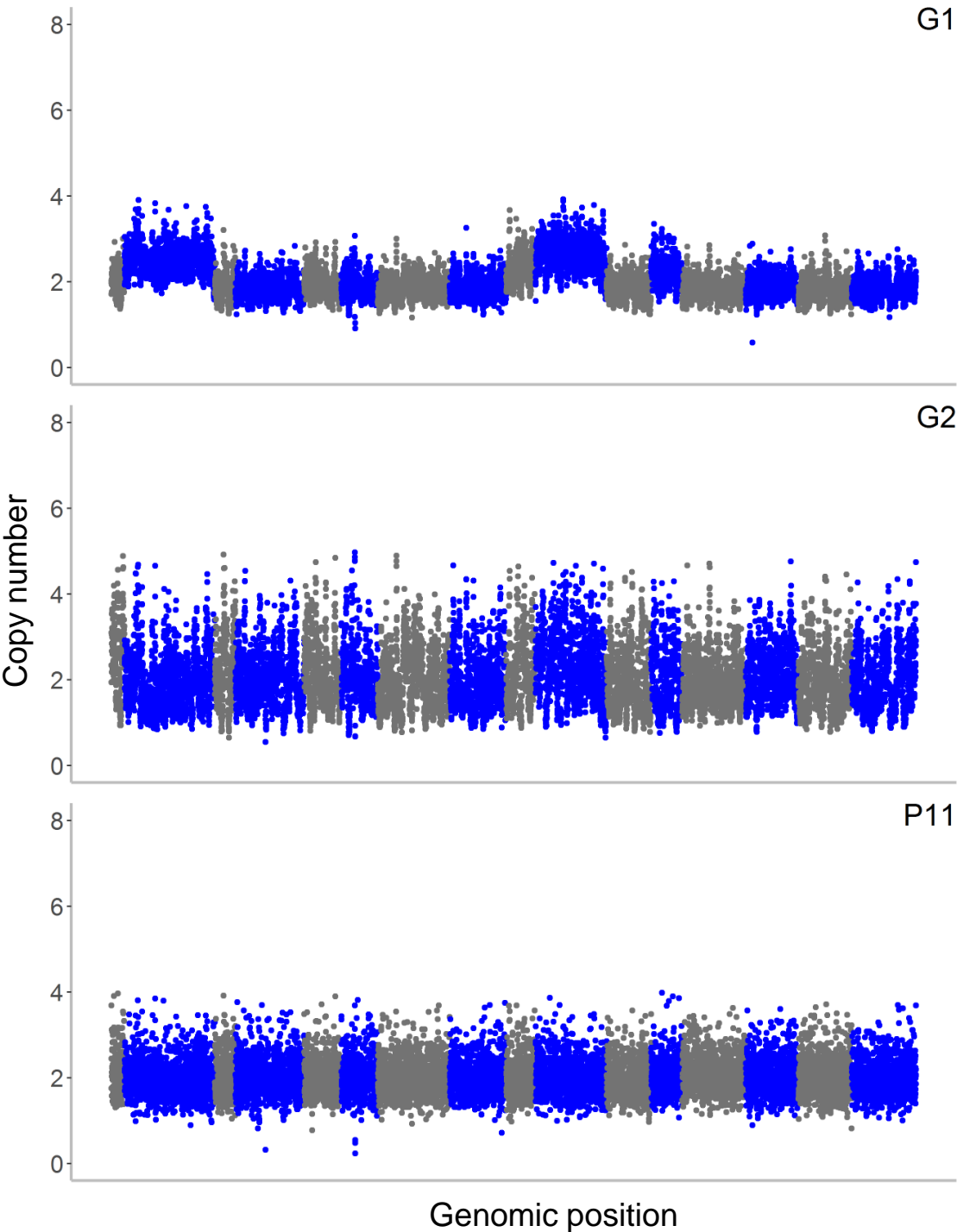

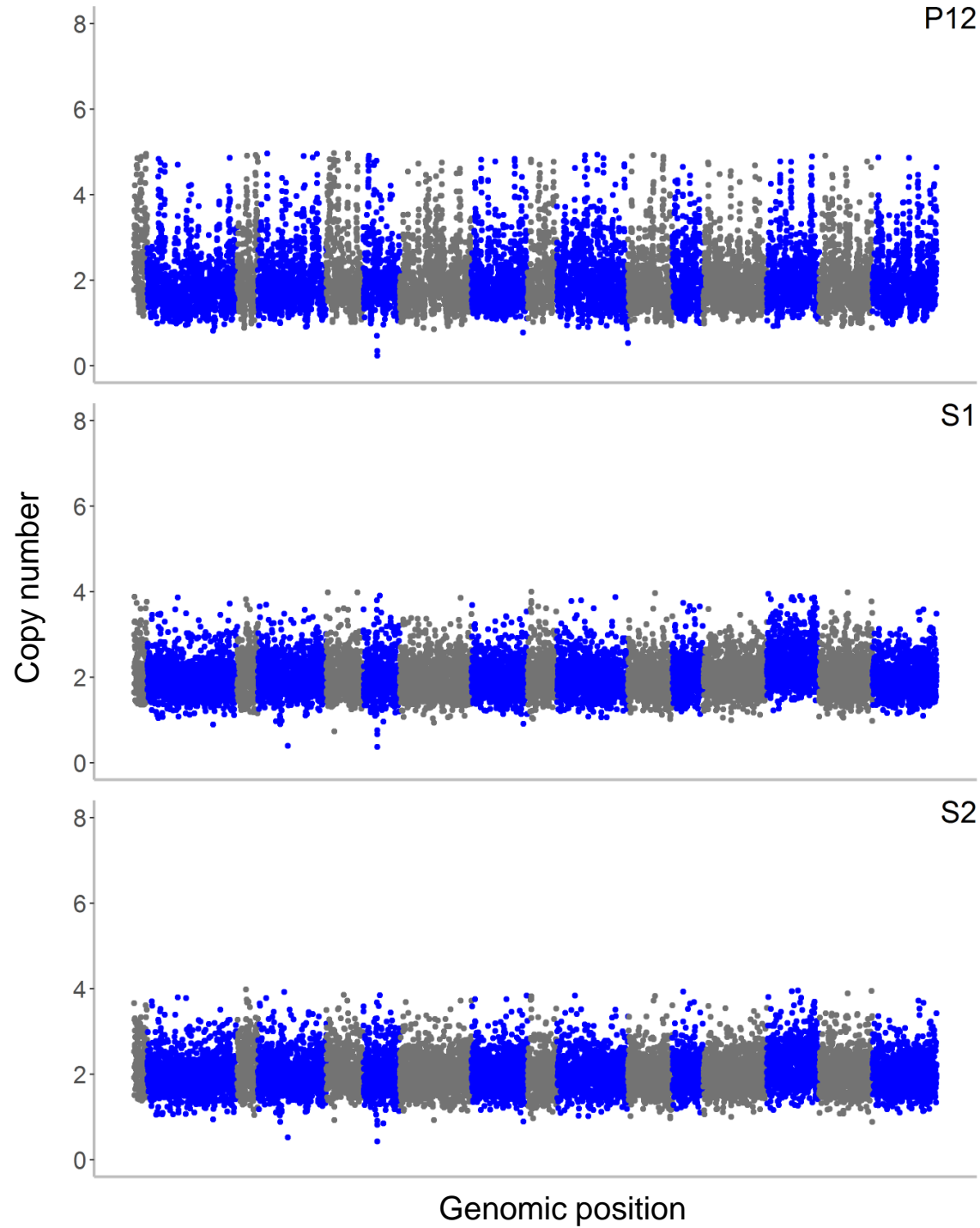

Supplement: S7 Fig — Copy number is plotted across the genome for S. uvarum evolved populations. Alternating grey and blue indicate different chromosomes (from chrI–chrXVI). Copy number was derived from average population sequencing read depth in 1000 bp intervals. Nutrient limitation is indicated in the upper right corner (G = glucose, S = sulfate, P = phosphate), numbers indicate independent populations. (PDF) [file pgen.1008383.s014.pdf]

Figure S9

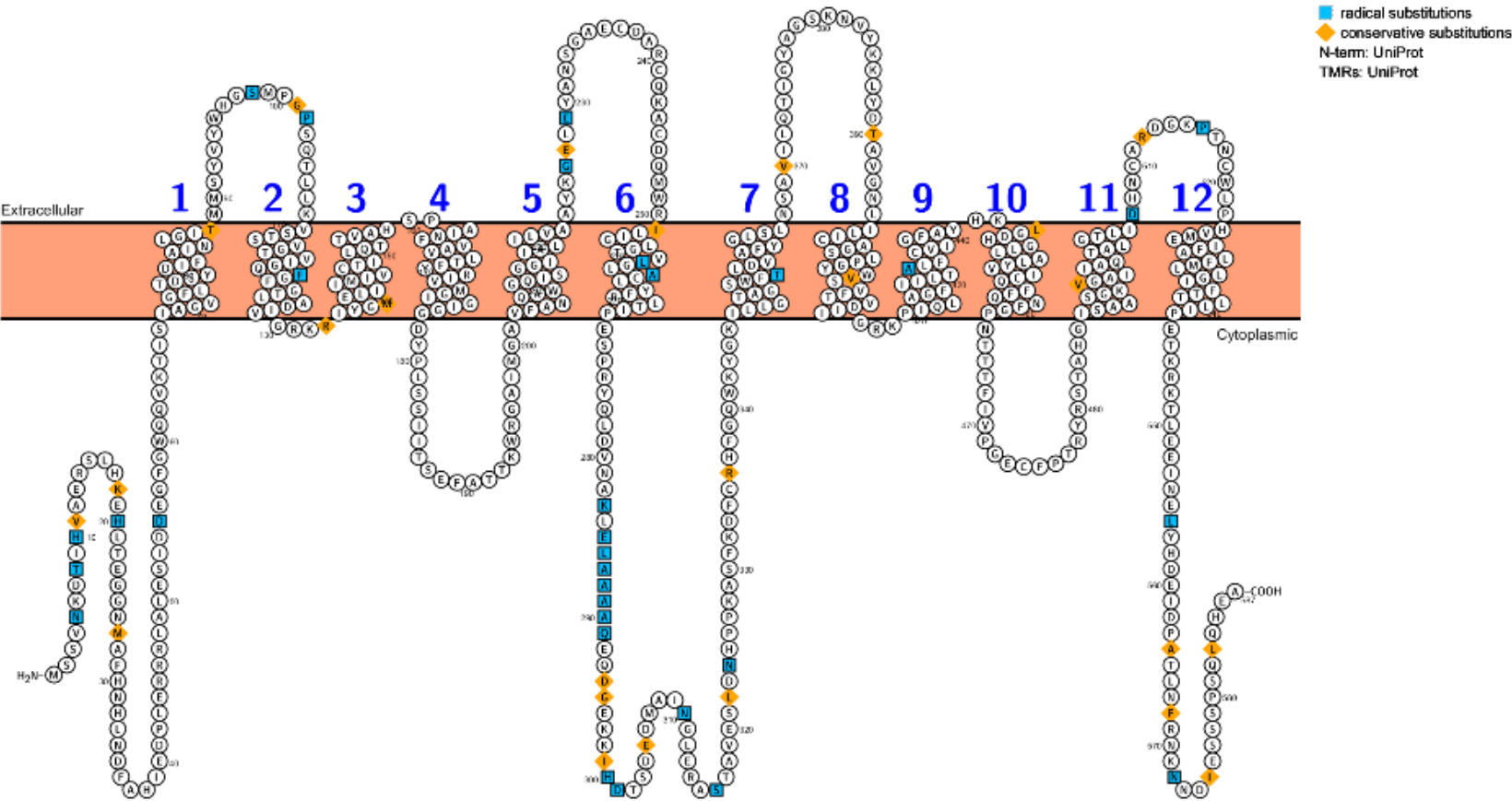

Supplement: S9 Fig — A 2D depiction of the structure of Pho84 was created by using UniProt protein accession P25297 (S. cerevisiae Pho84) with the program Protter [122]. Radical (blue square) and conservative (orange diamond) substitutions between S. cerevisiae and S. uvarum amino acids were annotated using a protein alignment (S8 Fig). (PDF) [file pgen.1008383.s016.pdf]

Figure S10

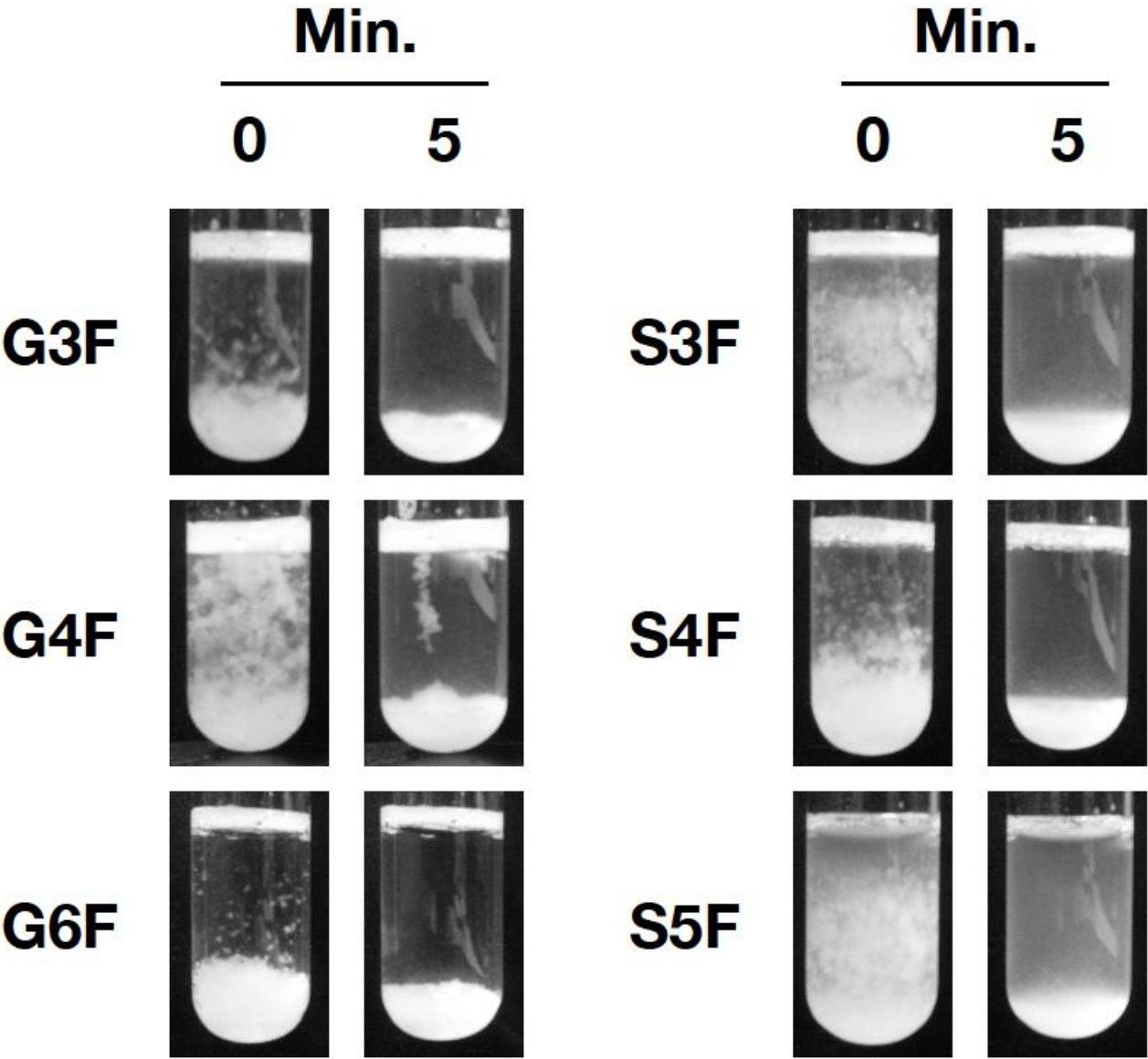

Supplement: S10 Fig — Overnight cultures were resuspended by vortexing for three seconds. Flocculent clones were photographed immediately after vortexing and then again after 5 minutes. (PDF) [file pgen.1008383.s017.pdf]
