# Supplementary material for: Temperature preference can bias parental genome retention during hybrid evolution
Source: PLoS Genet. 2019 Sep 16;15(9):e1008383. doi: 10.1371/journal.pgen.1008383 (PMC6762194; doi:10.1371/journal.pgen.1008383)
Supplement: S8 Fig — S. cerevisiae and S. uvarum alleles of Pho84 were aligned using Clustal Omega. Black indicates shared identity, white indicates a radical substitution, and grey indicates a conservative substitution. (PDF) [file pgen.1008383.s015.pdf]

```
S.cerevisiae    1 MSSVNKDTIHVAERSLHKEHLTEGGNMAFHNHLNDFAHIEDPLERRRLALESIDDEGFGW
S.uvarum        1 MSSVAKDNISIAERSLHRENLTEGGNLAFHNHLNDFAHIEDPLERRRLALESIDNEGFGW

S.cerevisiae   61 QQVKTISIAGVGFLTDSYDIFAINLGITMMSYVYWHGSMPGPSQTLLKVSTSVGTVIGQF
S.uvarum       61 QQVKTISIAGVGFLTDSYDIFAINLGISMMSYVYWHGDMPASSQTLLKVSTSVGTVIGQV

S.cerevisiae  121 GFGTLADIVGRKRIYGMELIIMIVCTILQTTVAHSPAINFVAVLTFYRIVMGIGIGGDYP
S.uvarum      121 GFGTLADIVGRKKIYGLELIIMIVCTILQTTVAHSPAINFVAVLTFYRIVMGIGIGGDYP

S.cerevisiae  181 LSSIITSEFATTKWRGAIMGAVFANQAWGQISGGIIALILVAAYKGELEYANSGAECDAR
S.uvarum      181 LSSIITSEFATTKWRGAIMGAVFANQAWGQISGGIIALILVAAYKNDLNYANSGAECDAR

S.cerevisiae  241 CQKACDQMWRILIGLGTVLGLACLYFRLTIPESPRYQLDVNAKLELAAAAQEQDGEKKIH
S.uvarum      241 CQKACDQMWRVLIGLGTVPGLLCLYFRLTIPESPRYQLDVNAELLR-VEKKEQEAEKKLY

S.cerevisiae  301 DTSDEDMAINGLERASTAVESLDNHPPKASFKDFCRHFGQWKYGKILLGTAGSWFTLDVA
S.uvarum      300 GTSDDDMAIHGLERAPTAVESIDMHPPKASFKDFCKHFGQWKYGKILLGTAGSWFMLDVA

S.cerevisiae  361 FYGLSLNSAVILQTIGYAGSKNVYKKLYDTAVGNLILICAGSLPGYWVSVFTVDIIGRKP
S.uvarum      360 FYGLSLNSAIILQTIGYAGSKNVYKKLYDSAVGNLILICAGSLPGYWMSVFTVDIIGRKP

S.cerevisiae  421 IQLAGFIILTALFCVIGFAYHKLGDHGLLALYVICQFFQNFGPNTTTFIVPGECFPTRYR
S.uvarum      420 IQLAGFIILTILFCVIGFAYHKIGDHGLLALYVICQFFQNFGPNTTTFIVPGECFPTRYR

S.cerevisiae  481 STAHGISAASGKVGAIIAQTALGTLIDHNCARDGKPTNCWLPHVMEIFALFMLLGIFTTL
S.uvarum      480 STAHGISAASGKIGAIIAQTALGTLINHNCAKDGKATNCWLPHVMEIFALFMLLGIFTTL

S.cerevisiae  541 LIPETKRKTLEEINELYHDEIDPATLNFRNKNNDIESSSPSQLQHEA
S.uvarum      540 LIPETKRKTLEEINEKYHDEIDPGTLNYRNKMNDVESSSPSQIQH--
```
